# Identifying neurobiological markers as predictors of antidepressant treatment using diffusion tensor imaging: A tract-based spatial statistical analysis of cingulate bundle

**DOI:** 10.1017/S1092852925000252

**Published:** 2025-09-01

**Authors:** Chunxia Yang, Jiaxin Han, Ning Sun, Penghong Liu, Kerang Zhang, Aixia Zhang, Zhifen Liu

**Affiliations:** 1 Department of Psychiatry, First Hospital of Shanxi Medical University, Taiyuan, 030001, P.R. China; 2 The First Clinical Medical College, Shanxi Medical University, Taiyuan, 030001, P.R. China; 3 Nursing College of Shanxi Medical University, Taiyuan, 030001, P.R. China

**Keywords:** Major depressive disorder, diffusion tensor imaging, tract-based spatial statistical analysis, SSRI, antidepressants response

## Abstract

It was found that a significant number of patients with major depressive disorder (MDD) did not respond to the treatment, leading to high ongoing costs and disease burden. The main objective of this study was to find neurobiological indicators that can predict the effectiveness of antidepressant treatment using diffusion tensor imaging (DTI). A group of 103 patients who were experiencing their first episode of MDD were included in the study. After 2 weeks of SSRI treatment, the group of patients was split into two categories: ineffectiveand effective. The FMRIB Software Library (FSL) was used for diffusion data preprocessing to obtain tensor-based parameters such as FA, MD, AD, and RD. Tract-Based Spatial Statistical (TBSS) voxel-wise statistical analysis of the tensor-based parameters was carried out using the TBSS procedure in FSL. We conducted an investigation to determine if there were notable variations in neuroimaging attributes among the three groups. Compared to HC, the effective group showed significantly higher AD and MD values in the left CgH. Correlating neuroimaging characteristics and clinical manifestations revealed a significant positive correlation between CgH-l FA and clinical 2-week HAMD-17 total scores and a significant positive correlation between CgH-r FA and clinical 2-week HAMD-17 total scores. Functional damage to the cingulum bundle in the hippocampal region may predispose patients to MDD and predict antidepressant treatment outcomes. More extensive multicenter investigations are necessary to validate these MRI findings that indicate treatment effectiveness and assess their potential significance in practical therapeutic decision-making.

## Highlights

1. DTI is a promising method for identifying neurobiological markers as predictors of antidepressant treatment.
2. TBSS analysis showed significantly higher AD and MD values of the left CgH in the effective group than in controls.
3. MDD was associated with reduced white matter integrity in the hippocampal subdivision of the cingulum tract.
4. Functional damage to the cingulum bundle in the hippocampal region may predict antidepressant treatment outcomes.

## Introduction

Major depressive disorder (MDD) is a highly prevalent disorder with an estimated lifetime prevalence of 16%.^1^ ^,^^2^ MDD is one of the leading causes of disability among individuals aged 15–44 years.^3^ ^,^^4^ While current treatments for MDD have demonstrated effectiveness, only 60% of patients respond to initial treatment, and even fewer achieve remission.^5^ ^–^^7^ Approximately one-third of patients with depression remain treatment resistant and do not respond to multiple pharmacological treatment regimens.^1^ ^,^^8^ Non-responsiveness of patients with MDD toward treatment rendered accounts for a substantial proportion of ongoing costs and disease burden.

It is challenging to predict the efficacy of primary treatment rendered, which results in delayed initiation of secondary treatment. This can lead to prolonged disease duration and mortality. Although clinical assessment is the cornerstone of management of patients with MDD, there is currently no consensus on pretreatment clinical predictors.^7^ ^,^^9^ Furthermore, depression symptomatology is heterogeneous and does not reflect a homogeneous biological and clinical entity.^10^ ^–^^12^ The shortcomings of current standard clinical measures have led to the recent focus on the development of novel mechanism-based biomarkers that reflect disruptions in fundamental brain circuits.^13^ In recent decades, a multitude of neuroimaging studies have been dedicated to investigating the pathogenesis of MDD. However, there remains limited understanding of the pathophysiological basis for variations in patient response to antidepressant medication and difficulties in rehabilitation.^14^ Moreover, the simultaneous quantification of treatment-responsive patterns in both structural connectivity and functional connectivity has been demonstrated to capture distinct connectivity related to the response to antidepressant treatment.^15^ Advances in neuroimaging have provided new insights into the functional and structural brain changes in patients with MDD, which aid in further identification of the subtypes of depression with different treatment responses or disease differences.^16^ ^,^^17^ Therefore, the use of magnetic resonance imaging (MRI) to characterize brain differences between drug responders and non-responders may improve patient treatment and outcomes. Characterizing the organization and integrity of white matter in the brain using diffusion tensor imaging (DTI) may provide a means to distinguish between antidepressant responders and non-responders. Several DTI measures of altered white matter specifically distinguish medication responders and non-responders at baseline and show promise for predicting treatment responses in patients with MDD.^18^ ^–^^20^

Recent DTI studies have revealed that the structural white matter connectivity of neural circuitry distinguishes patients with MDD from their healthy peers. These studies have found that different segments of the anterior cingulate cortex (ACC) represent important hubs in these networks. In addition, the rostral ACC is connected to the frontostriatal reward network, which plays an important role in the pathogenesis of MDD.^21^ White matter alterations associated with these connections in the reward circuit have been found to be associated with depression and the risk of developing depression. The cingulum bundle (CB) is a critical white matter tract that runs from the subgenual cingulate to the occipital lobe adjacent to the corpus callosum.^3^ Altered CB white matter has been identified in patients at high risk for depression and is also associated with subclinical symptoms.^22^ The cingulum bundle conveys a substantial portion of projections originating from the ACC towards regions that play a critical role in the pathophysiology of depression, such as the hippocampus and the parahippocampal gyrus.^23^ ^,^^24^ In this study, we have identified two specific white matter fiber tracts related to the amygdala and subgenual ACC as potential predictors of treatment outcomes. The CB is an association pathway innervated by the cingulate gyrus and connected to the hippocampus [subdivided into cingulate (CgC) and hippocampal (CgH) parts].^25^

In order to optimize the translational potential, we used a technique called tract-based spatial statistical (TBSS) analysis. This technique is a highly reproducible, robust, and easily automated method for generating simple metrics of white matter tracts.^26^ The structural connectivity and functional connectivity strength and persistence between different nodes in the brain are all influenced by the structural and microstructural properties of white matter.^27^ DTI can quantify the restriction of free-moving water molecules in brain tissue, where indices such as fractional anisotropy (FA), mean diffusivity,^28^ radial diffusivity (RD), and axial diffusivity (AD) describe the microstructural properties of white matter.^29^ ^–^^31^ These measures have proven to be useful in differentiating between clinical and neurotypical groups. FA is the most widely used parameter of the DTI metric and provides a summary estimate of the degree to which tissue micro- and macro-organization causes diffusion anisotropy. FA is sensitive to microstructural changes in the white matter, and higher values are associated with increased integrity. FA indicates various characteristics of axon fibers, including the number and size of axon fibers along with the density of crossing fibers.^32^ However, FA is not specific to any particular source of change,^33^ and some authors urge caution against overinterpreting the anisotropy results.^34^ ^,^^35^ MD measures the overall diffusion restriction in a voxel and is sensitive to disruption of either axonal or myelin integrity.^36^ Previous studies have suggested that AD is sensitive to axonal pathologies and RD to myelination,^37^ which could reflect the white matter tissue microstructure. It has been interpreted as an index of the magnitude of fluid viscosity and cellularity sensitivity.^35^ ^,^^38^ Therefore, AD, RD, and MD values may complement FA values and help interpret potential underlying tissue microstructural alterations.^39^ ^,^^40^ However, it remains unclear how these characteristics correlate with MDD, whether they are associated with more severe persistent depressive experiences, or whether they can reflect predictors of antidepressant treatment. Thus, in the present study, by examining FA together with MD, RD, and AD, it was possible to derive a more comprehensive mapping of neurobiological variations in MDD.

Herein, we present the first planned analysis to evaluate the predictive utility of DTI. We also tested whether these tracts were significantly different between patients with MDD (MDD group) and controls (control group). Our primary objective was to investigate baseline white matter differences in two fiber tracts (CgC and CgH) associated with response or non-response to SSRI (selective serotonin reuptake inhibitor) following 2 weeks of treatment with antidepressants in patients with MDD. We hypothesized that the tracts that predict outcomes are also altered in patients with MDD in comparison to healthy controls (HC) at pretreatment baseline. We investigated the temporal changes in DTI metrics associated with a favorable medication response during the 2-week study period. The findings would lead to an increasing interest in the neurobiological underpinnings of the pathophysiology of MDD.

## Materials and methods

### Collection and evaluation of clinical data

#### Selection of the study participants

Between September 2009 and December 2018, a study conducted at the Mental Health departments of the First Hospital of Shanxi Medical University recruited a group of 103 patients with MDD. These patients were experiencing their first episode of MDD and had not undergone any prior treatment for the condition. The recruitment process involved both inpatient and outpatient departments. The detailed process of collection and diagnosis for subjects was described previously by Zhang where 101 patients with MDD and 53 controls were included.^41^ The patients were also assessed with the Chinese Version of the Modified Structured Clinical Interview for DSM-IV TR Axis I Disorders Patient Edition (SCID-I/P, 11/2002 revision). The diagnosis of MDD was independently confirmed by at least two consultant psychiatrists in accordance with the criteria outlined in the Diagnostic and Statistical Manual of Mental Disorders, Fourth Edition (DSM-IV). All patients underwent an initial evaluation that involved utilizing MRI scans and assessing their mental health using standardized rating scales, such as the Hamilton Depression Rating Scale (HAMD-17) for depressive symptoms and the Hamilton Anxiety Rating Scale (HAMA) for anxiety symptoms. To investigate neurobiological indicators for MDD, a cohort of 38 individuals without any familial ties to the patients, matched for age, gender, and education level, and in good health, was selected from both the local community and the university. All the attendees granted their consent after being informed.

Patients of MDD from Han Chinese ethnic group underwent diagnosis and screening through the application of specific inclusion criteria by two psychiatrists who possessed ample experience in the field: (1) age range from 18 to 60 years old; (2) right-handed; (3) the patient, who had not received any treatment, was diagnosed with an initial occurrence of MDD following the DSM-IV guidelines; (4) the HAMD-17 score is greater than 17 and the HAMA-14 score is less than 14; and (5) the participants in this study were only included if they had given informed consent.

They were not included if they had either MDD or bipolar disorder caused by organic diseases or antipsychotic drugs: (1) head trauma with loss of consciousness, neurological illness; (2) concomitant additional Axis I psychiatric disorders; (3) severe organic conditions are inclusive of neurological disorders, major liver and kidney impairment, cardiac ailments, and cranial injuries; (4) the individual presents with indicators such as intense thoughts of self-harm and suicide, a previous record of suicide attempts (scoring ≥2 on the HAMD-17 scale), clear impulsive behavior, or a tendency to be uncooperative.; (5) breastfeeding or pregnant women; and (6) individuals having conditions that prohibit them from undergoing an MRI scan.

#### Treatments rendered to the patients

After being enrolled, patients with untreated first-episode MDD received standardized antidepressant medications. These drugs belong to the class of SSRIs, including escitalopram tablets (Janssen; 5–20 mg/day), fluoxetine dispersible tablets (Eli Lilly; 10–40 mg/day), citalopram tablets (Envac; 10–40 mg/day), and sertraline tablets (Pfizer; 25–200 mg/day).

All patients were given low doses of medications, and their dosage was adjusted based on their condition. Patients with sleep disorders received either short-term benzodiazepines or supportive psychotherapy, depending on their needs. No other treatments, such as antidepressants, antipsychotics, electroconvulsive therapy, or physical therapies, were administered during the 2-week treatment period. The patients’ symptoms were assessed and documented prior to treatment initiation and again after 2 weeks.

#### Clinical data collection

##### General demographic information

The case report form (CRF) created by our department was used to record the participants’ demographic information, such as gender, age, educational attainment, marital status, smoking and alcohol habits, substance abuse, and family background.

##### Scales for clinical symptoms

For the evaluation of depression symptoms, HAMD-17 was employed, whereas HAMA was used for assessing anxiety symptoms.

### Diffusion MRI data acquisition

Diffusion MRI scans were performed using A MAGNETOM Trio Tim 3.0 T and a 12-channel phased array surface head coil (Siemens Medical Solutions, Germany). All the participants underwent MRI after being fully informed of the procedure. During the scan, the participants were instructed to remain awake, lie flat at rest, breathe calmly, and keep their heads fixed. High-resolution T1-weighted anatomical images were obtained. The participants were also given an alarm bell to end the scan if they felt uneasy. High-resolution transaxial T1-weighted anatomical images for Voxel-Based Morphometry (VBM) were obtained using a 3D-FLASH sequence. Subsequently, a 10-minute DTI scan was acquired utilizing a single-shot echo planar imaging sequence with the specified parameters. DTI was collected with a single spin echo planar imaging sequence; axial scanning; scanning a total of 45 continuous levels; 12 diffusion sensitive gradient directions; diffusion sensitive coefficient, b = 1000; axis scan for best tonsure diffusion weighted imaging, b = 0; TR (repetition time) = 3600 ms; TE (echo time) = 90 ms; matrix = 128*128; vision = 24*24 cm; corner being 90; layer= 0 mm; and scanning time of 4 min 14 s.

### DTI data preprocessing

In this study, we utilized the FMRIB Software Library (FSL) (www.fmrib.ox.ac.uk/fsl)^42^ to preprocess the diffusion data, along with tensor-based parameters such as FA, MD, AD, and RD parameters, which were obtained from TBSS to extract relevant data.^26^ The TBSS procedure in FSL was employed to perform voxel-wise statistical analysis of the tensor-based parameters using tract-based spatial statistical analysis.

Initially, the data of each participant were pre-processed using FSL software. This involved performing head-motion eddy current correction and gradient direction correction to acquire the brain range mask. Domain: Software and data processing; background: preprocessing data for analysis; revised sentence: To obtain the brain range mask, the FSL software was utilized to preprocess the data of each participant, which involved correcting head-motion eddy currents and gradient directions. The autoPtx software was employed to compute the dispersion index (for obtaining the FA, MD, AD, and RD indices), predict the orientation distribution of BedPostX, and align the DTI space with the standard space.^43^ ^–^^45^

### Statistical analysis

After excluding participants with invalid or unreliable data, a total of 101 individuals diagnosed with MDD and 53 individuals without any known mental health conditions were recruited for this research analysis. The statistical analysis in MATLAB involved performing a Univariate ANOVA on three groups, resulting in the calculation of p-values and their corresponding F-values. A follow-up analysis was conducted to identify statistically significant differences among the main groups. A post hoc test was subsequently performed to identify significant main group differences. Statistical significance was set at p < 0.05, corrected by the LSD test.

Furthermore, Pearson’s correlation analysis was conducted to evaluate any relationships between neuroimaging characteristics and clinical presentations (clinical 2-week HAMD-17 total scores). The results were considered statistically significant at p < 0.05.

## Results

### General demographic data and clinical characteristics

We conducted an analysis, including 103 patients with MDD and 38 individuals without any mental health conditions as HC. The data used for this analysis were collected through diffusion MRI. The data in Table 1 present the demographic and clinical features of individuals with MDD alongside a group of healthy individuals. The independent sample t-test and chi-square test revealed no significant variances in age, sex, and level of education between the two groups.

**Table 1.** General Demographic Information and Clinical Characteristics of the Patients with MDD and Normal Controls

Variables	MDD patients (n = 103)	HCs (n = 38)	p-value
Age, years ( 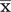 ± s)	30.05 ± 10.15	32.18 ± 8.07	0.245^a^
Sex (F/M)	67/36	20/18	0.181^b^
Education, years ( 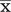 ± s)	14.56 ± 3.06	13.87 ± 2.87	0.226^a^
HAMD–17 scores ( 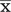 ± s)	20.9 ± 3.53	NA	-

a t-test.

b χ2 test.

Following a 2-week course of SSRI medication, the individuals undergoing treatment were classified into two groups, ineffective (n = 23) and effective (n = 28) groups, according to their HAMD-17 scores. Table 2 outlines the demographic and clinical attributes of both the ineffective and effective groups, encompassing general population data. Based on the outcomes of statistical tests such as the independent sample t-test and chi-square test, there were no significant differences between the two groups regarding age, sex, level of education, and HAMD-17 scores.

**Table 2.** General Demographic Data and Clinical Characteristics of the Ineffective Group and the Effective Group

Variables	The effective group (n = 28)	The ineffective group (n = 23)	HCs (n = 38)	p-value
Age, years ( 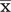 ± s)	34.64 ± 10.10	30.00 ± 9.81	32.18 ± 8.07	0.203^a^
Sex (F/M)	19/9	13/10	20/18	0.457^b^
Education, years ( 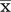 ± s)	14.07 ± 3.01	15.30 ± 3.72	13.87 ± 2.87	0.208^a^
HAMD–17 scores (0 wk) ( 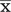 ± s)	21.25 ± 3.40	20.48 ± 2.84		0.450^a^
HAMD–17 scores (2 wk) ( 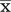 ± s)	11.43 ± 2.01	18.96 ± 3.71		<0.001^a^

a t-test.

b χ2 test.

### DTI data results

**Figure 1.**
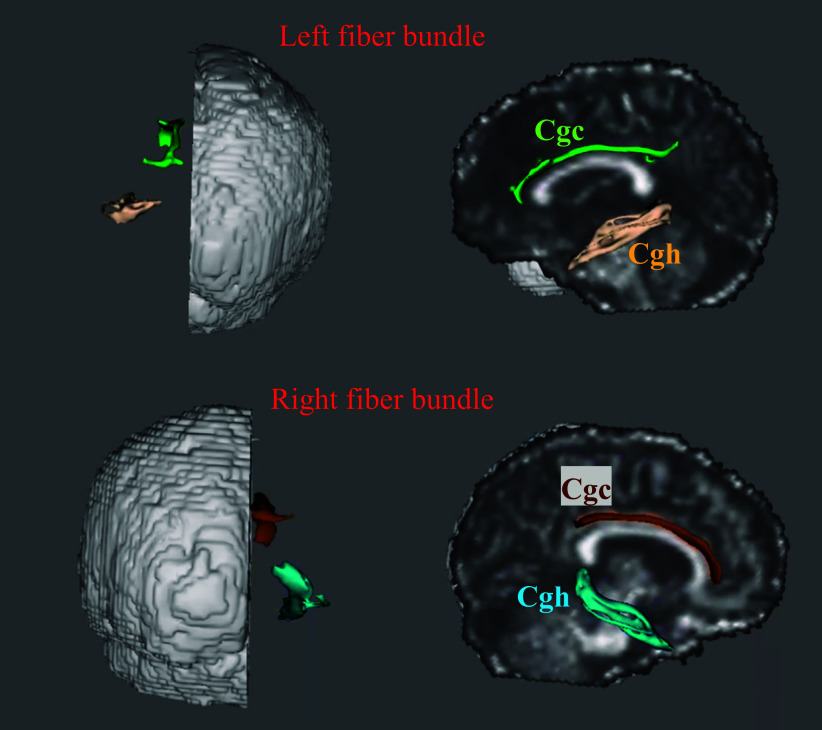
Example of the cingulate gyrus part of the cingulum and the parahippocampal part of the cingulum. A: Left CgC and CgH. B: Right CgC and CgH.

**Figure 2.**
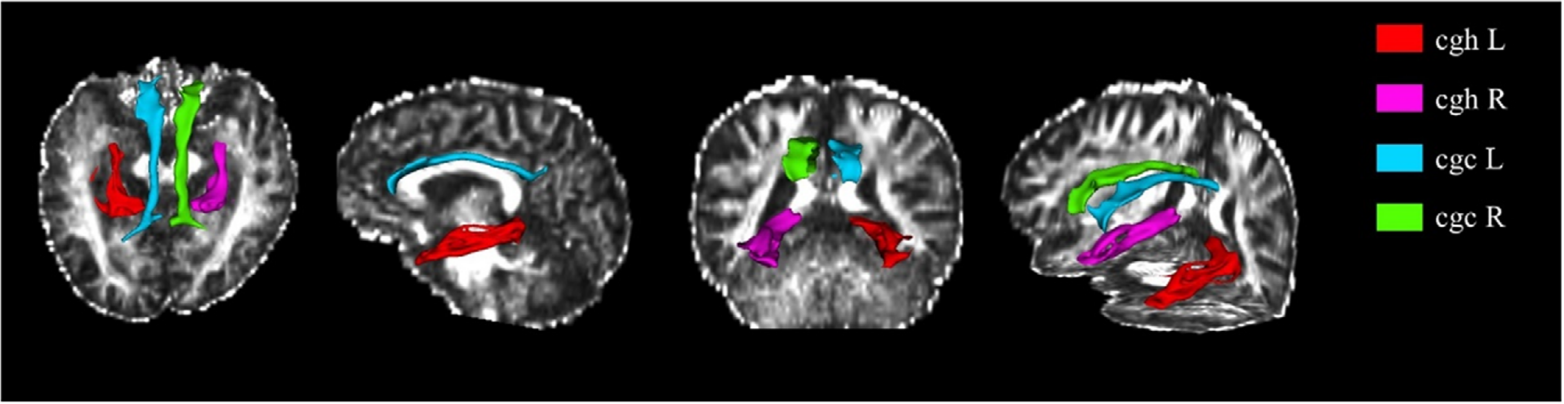
Anatomical location visualization of CgC and CgH in the cingulate tract.

The CgC and CgH divide the right and left brain, resulting in four fibrous tracts. A fiber probability map with a card threshold of 0.001 and a fiber bundle mask were obtained (Figures 1 and 2).

### TBSS analysis

Tracts spanning the anterior corpus callosum to the cingulate bundle were analyzed. Probabilistic fiber tracking was performed to obtain the fibers of interest. The CgC and CgH divide the right and left brain, resulting in four fibrous tracts. A fiber probability chart with a card threshold of 0.001 was obtained from the general tracing results, and a fiber bundle mask was obtained. The average FA, MD, AD, and RD index values within the mask range of the four fiber bundles for each participant were then extracted. There were significant differences in AD and MD values with TBSS analysis in CgH-l among the effective, ineffective, and HC groups (p < 0.05; Table 3).

**Table 3.** Neuroimaging Characteristic Differences of the Responsive, Unresponsive Groups, and Health Control Subjects at Baseline

		The ineffective group (n = 23)	The effective group (n = 28)	HC (n = 38)	F-value	p-value
FA						
CgC	L	0.31 ± 0.07	0.31 ± 0.09	0.31 ± 0.07	0.085	0.919
	R	0.31 ± 0.11	0.29 ± 0.09	0.31 ± 0.07	0.444	0.643
CgH	L	0.26 ± 0.06	0.24 ± 0.05	0.25 ± 0.05	0.665	0.517
	R	0.29 ± 0.07	0.26 ± 0.07	0.27 ± 0.06	1.881	0.159
MD ( 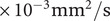 )						
CgC	L	1.04 ± 0.3	1.11 ± 0.31	1.13 ± 0.26	0.784	0.460
	R	1.03 ± 0.25	1.13 ± 0.34	1.12 ± 0.3	0.800	0.453
CgH	L	0.97 ± 0.17	1.03 ± 0.16	0.92 ± 0.17	3.581	0.032
	R	0.98 ± 0.19	1.05 ± 0.32	0.96 ± 0.23	1.047	0.356
AD ( 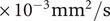 )						
CgC	L	1.36 ± 0.34	1.46 ± 0.33	1.47 ± 0.27	0.882	0.417
	R	1.35 ± 0.29	1.44 ± 0.35	1.46 ± 0.32	0.940	0.395
CgH	L	1.2 ± 0.19	1.27 ± 0.19	1.14 ± 0.18	4.150	0.019
	R	1.26 ± 0.19	1.3 ± 0.34	1.19 ± 0.24	1.303	0.277
RD ( 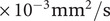 )						
CgC	L	0.88 ± 0.29	0.94 ± 0.31	0.97 ± 0.26	0.682	0.508
	R	0.87 ± 0.25	0.97 ± 0.35	0.95 ± 0.3	0.714	0.493
CgH	L	0.85 ± 0.17	0.91 ± 0.16	0.81 ± 0.18	3.065	0.052
	R	0.85 ± 0.19	0.92 ± 0.31	0.84 ± 0.23	0.968	0.387

**Figure 3.**
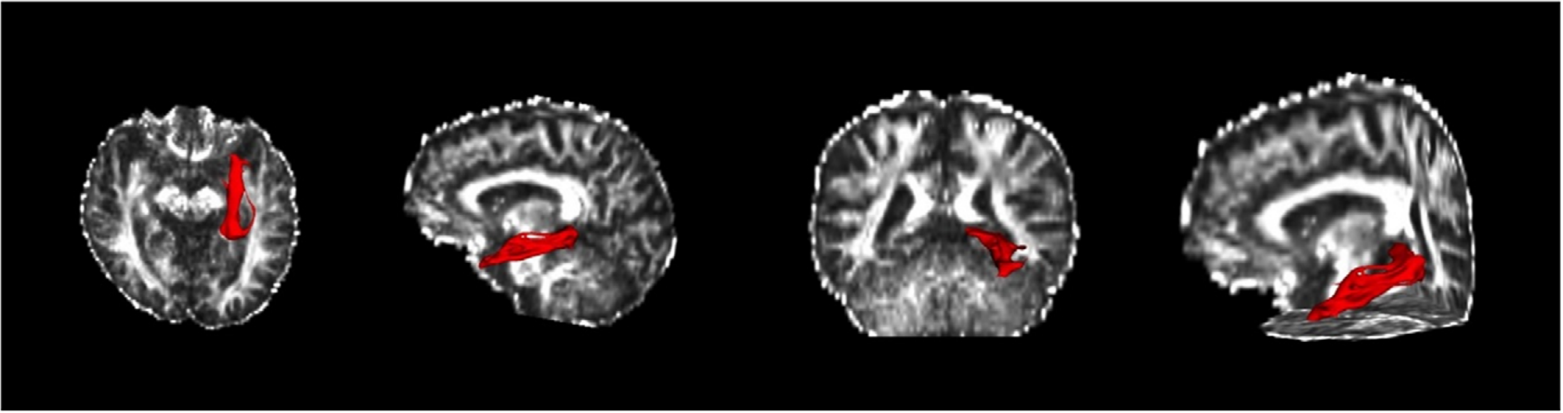
Anatomical location visualization of the left CgH with significant differences.

A post hoc test was subsequently performed to identify significant main group differences. Compared to HC, the effective group showed significantly higher AD and MD values in the left CgH (p < 0.05; Figure 3, Tables 4 and 5).

**Table 4.** Significant Differences in Neuroimaging Characteristics (AD) of CgH_l Among the Responsive, Unresponsive Groups, and Health Control Subjects

Regions	t	p value
The effective group VS the ineffective group	−1.241	0.221
The effective group VS HCs	2.886	0.005
The ineffective group VS HCs	1.374	0.175

**Table 5.** Significant Differences in Neuroimaging Characteristics^28^ of Cgh l Among the Responsive, Unresponsive Groups, and Health Control Subjects

Regions	t	p value
The effective group VS the ineffective group	−1.443	0.155
The effective group VS HCs	2.695	0.009
The ineffective group VS HCs	0.993	0.325

### Correlating analysis

**Figure 4.**
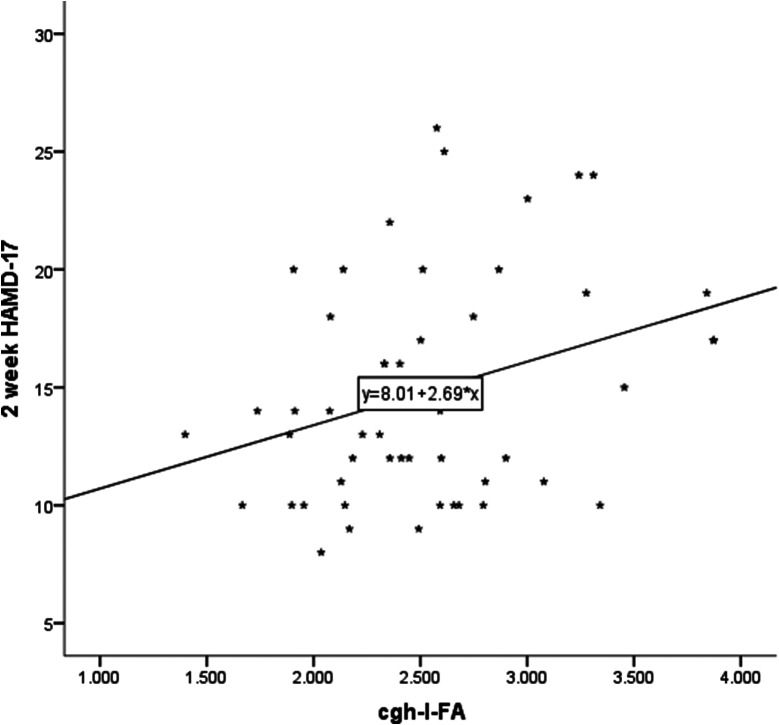
Correlation between CgH-l FA and clinical 2-week HAMD-17 total scores.

**Figure 5.**
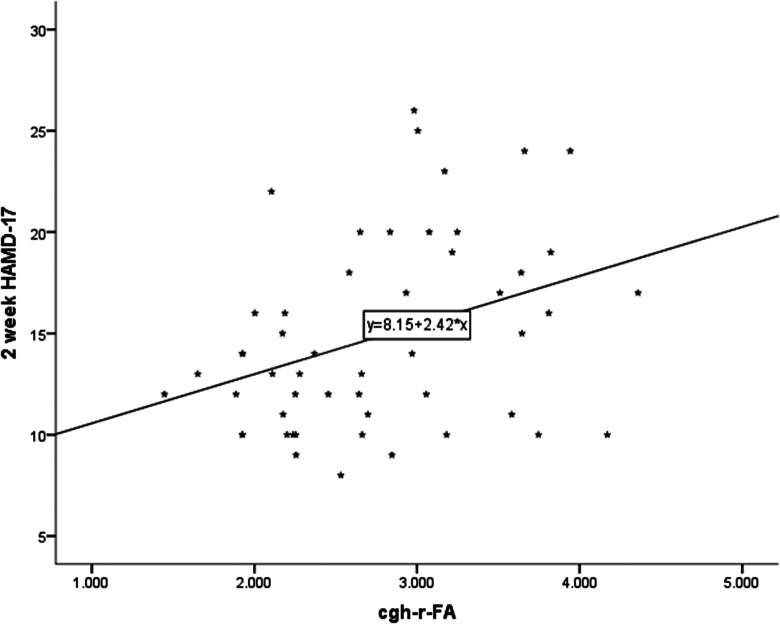
Correlation between CgH-r FA and clinical 2-week HAMD-17 total scores.

We performed Pearson’s correlation analysis to evaluate potential associations between 16 neuroimaging features and clinical manifestations(clinical 2-week HAMD-17 total scores) Correlating neuroimaging characteristics and clinical manifestations revealed a significant positive correlation between CgH-l FA and clinical 2-week HAMD-17 total scores (r = 0.320, p = 0.022) and a significant positive correlation between CgH-r FA and clinical 2-week HAMD-17 total scores (r = 0.358, p = 0.010). There were no significant correlations between the other 14 neuroimaging characteristics and clinical manifestations (Figures 4 and 5).

## 4 Discussion

To the best of our knowledge, this is the first study using tractography to evaluate DTI-MRI parameters in the cingulum brain white matter tract of patients with MDD to assess the response toward anti-depressants being administered. These potential implications and areas for future studies are discussed in the following sections. Our findings revealed a general pattern among the ineffective, effective, and control groups. Two main findings were obtained. First, significantly higher AD and MD values of the left CgH were observed in patients with MDD than in controls. There was a trend towards higher RD values in the left CgH in patients with MDD (p = 0.052). Second, correlations between neuroimaging characteristics and clinical manifestations were analyzed. There was a significant positive correlation between CgH-l FA and HAMD-17 total scores (after 2 weeks), and a significant positive correlation between CgH-r FA and HAMD-17 total scores (after 2 weeks). Exploring clinical presentations underlying neuronal networks may advance our pathophysiological understanding of MDD.^12^ ^,^^46^

The cingulate is a collection of white matter fibers that project from the cingulate to the entorhinal cortex of the brain and is used for communication between the components of the limbic system. Cingulate fibers connect to various structures, including the parahippocampal cortex.^40^ ^,^^47^ It has been shown that the function of this network is damaged in depression^48^ and may normalize with the administration of antidepressants.^49^ ^,^^50^ In particular, the CB represents a major link within the connectivity core of the brain.^51^ ^,^^52^ Pathological changes within the CB may impact mood regulation in individuals diagnosed with MDD. CB is a critical white matter tract that surrounds the corpus callosum and connects the ipsilateral subcortical nuclei, cingulate gyrus, and areas of the frontal, temporal, and parietal lobes.^22^ The CB runs from the subgenual cingulate to the occipital lobe adjacent to the corpus callosum.^3^ ^,^^22^ Due to the comprehensive and integrating role of the CB, several previous studies have assumed that the CB microstructure is associated with several symptoms of depression and therefore reflects overall depression severity.^46^ According to the ICBM-DTI-81 atlas, the cingulum is divided into CgH inferiorly and CgC superiorly at the level of the splenium of the corpus callosum.^5^ In support of our hypotheses, patients with MDD showed increased MD and AD values in the left CgH in comparison to HCs. The effective group showed higher MD and AD values for the parahippocampal subdivision of the cingulum than the ineffective group after 2 weeks of SSRI treatment.

Our research contributes to the wide range of research on changes in neuroimaging features in individuals diagnosed with MDD.^53^ In this study, we examined disruptions to neuroimaging features in individuals who were responsive and unresponsive to treatment, as well as in HC. We also investigated the relationship between these disruptions and the current severity of depression. Our findings showed an overall increase in MD accompanied by higher AD and RD (p = 0.052) in patients with MDD, which were consistent with recent DTI findings in depressed individuals.^53^ The value of AD indicates the extent of water diffusion along the white matter pathways.^54^ The observed negative correlation between AD and neuroticism may be understood as the outcome of heightened reactivity to stress in individuals who consistently encounter negative emotions. Based on prior research, our study observed increased AD and MD values in the left CgH in patients; this may indicate a decline in the microstructure of white matter among individuals with MDD.^55^ The results additionally validate the deterioration of the white matter microstructure in patients with MDD. The hippocampus, being part of the limbic system, plays a role in the perception of emotions and behavior related to reward.^56^ Furthermore, it displays cortical thinning in patients with MDD.^57^

Correlations between neuroimaging characteristics and clinical manifestations were considered, revealing a significant positive correlation between CgH-l/R FA and HAMD-17 total scores after 2 weeks of clinical treatment. Earlier investigations have suggested that the remission of depressive disorders is associated with increased FA values in the right cingulum.^58^ It is hypothesized that the CB microstructure associated with this association might serve as an indicator of depression severity. We speculate that increased ACC activity, an early marker associated with the response to antidepressant treatment, induces such neuroplastic processes.^59^ ^–^^62^ Nevertheless, to reach conclusive findings on this matter, it is imperative to conduct longitudinal studies that include control groups. An additional animal experiment conducted on WKY rats provided further evidence of reduced white matter integrity in both the corpus callosum and cingulum. This was established through a detailed voxel-by-voxel analysis, which indicated higher values for MD, DR, and DA, along with lower values for FA.^63^ A different investigation found that patients with MDD exhibit reduced FA and increased MD in the corpus callosum compared to controls.^64^ A study conducted by other researchers found that patients with depression have lower levels of FA in the left cingulate cortex compared to individuals without depression.^65^ Furthermore, it has been observed that elevated diffusivity levels in the hippocampal cingulum are associated with the presence of more severe depressive symptoms

Our results also support the role of the combination model of significantly decreased FA values, increased AD and MD values of the CgH in predicting antidepressant treatment outcomes. DTI is a highly promising technique used to assess alterations in brain tissue structure caused by various neuropathological conditions and therapies, such as MDD. Applications of DTI are used to examine the pathological features of white matter (eg ischemia, myelination, axonal damage, inflammation, and edema).^5^ Given the current uncertainty surrounding the interpretation of DTI parameters in neuropathology, FA is commonly understood as a composite indicator reflecting both axon density and myelin content. This measure is remarkably responsive to minute alterations in the microstructure, although it lacks specificity in identifying the particular nature of the changes (eg radial or axial).^66^ We utilized various diffusion tensor measures (MD, AD, and RD) to enhance precision and provide a more accurate representation of the tissue microstructure. According to prior studies, the progressive shrinkage of the hippocampus may be associated with the progressive rupture of the cingulum bundle. The alterations in brain regions may stem from the diverse impacts of a shared pathological process, while the loss of gray matter could cause disruptions in the connectivity of white matter tracts.^67^ Although making specific conclusions about the neurobiological origins of variations in diffusion measures is challenging, this observed pattern aligns with findings from prior studies on the risk of depression.^68^ Furthermore, the TBSS approach has demonstrated a high sensitivity in examining white matter integrity through scalar measures such as FA, MD, RD, and AD across various neuropsychiatric disorders.^30^ Despite the robustness of our findings, it is important to acknowledge potential limitations of TBSS in fully characterizing all voxels specific to a tract.^69^

While acknowledging the reliability of the anticipated outcomes, it is crucial to take into account certain constraints. Initially, the investigation was carried out within a relatively limited time frame. There *is* a possibility that the variability of the WM may become apparent on a larger time scale, such as during a 6-month visit period.^58^ However, we used baseline MRI to predict that the difference of 2 weeks can embody the early tendency of plasticity in WM alterations. Furthermore, it is important to note that while the sample size of this particular study was larger than that of previous studies, it is still necessary to replicate these findings in an independent cohort to ensure their generalizability. Our results demonstrated substantial effect sizes and were consistent across both assessment periods, despite the limitation of a small sample size. Third, DTI is presently the sole technique with the aptitude to chart the structural arrangement of fibers in vivo.^47^ Nevertheless, the outcomes need to be approached with care. Fourth, our knowledge about the neurobiological basis of changes in FA, AD, MD, and RD in the cingulum related to both MDD and response to treatment was limited. It has been demonstrated that the duration of untreated depression is associated with increased volume loss in some specific brain regions. This aspect will undoubtedly be a focal point in our forthcoming research endeavors. Further research conducted across multiple medical centers is essential to validate the MRI associations with patient treatment response and to evaluate their potential impact on clinical decision-making in real-world practices.

## Conclusions

Baseline DTI scans of invariant scalar metrics associated with axonal integrity have been shown to distinguish antidepressant responders, non-responders, and controls in the parahippocampal part of the cingulum. Our findings are consistent with those of previous studies that have identified an association between MDD and reduced white matter integrity in the hippocampal subdivision of the cingulum tract. Functional damage to the cingulum bundle in the hippocampal region may predispose patients to MDD and predict antidepressant treatment outcomes.
